# Study of Time-Resolved Dynamics in Turbid Medium Using a Single-Cavity Dual-Comb Laser

**DOI:** 10.1021/acsphotonics.4c00254

**Published:** 2024-06-05

**Authors:** Binbin Zhang, Christopher Phillips, Esteban Venialgo Araujo, Sophinese Iskander-Rizk, Justinas Pupeikis, Benjamin Willenberg, Ursula Keller, Nandini Bhattacharya

**Affiliations:** † Department of Precision and Microsystems Engineering, Delft University of Technology, Mekelweg 2, Delft 2628 CD, The Netherlands; ‡ Department of Physics, Institute for Quantum Electronics, ETH Zurich, Zurich CH-8093, Switzerland

**Keywords:** diffusing-wave spectroscopy, blood flow, near-infrared spectroscopy, time-of-flight, single-photon detection

## Abstract

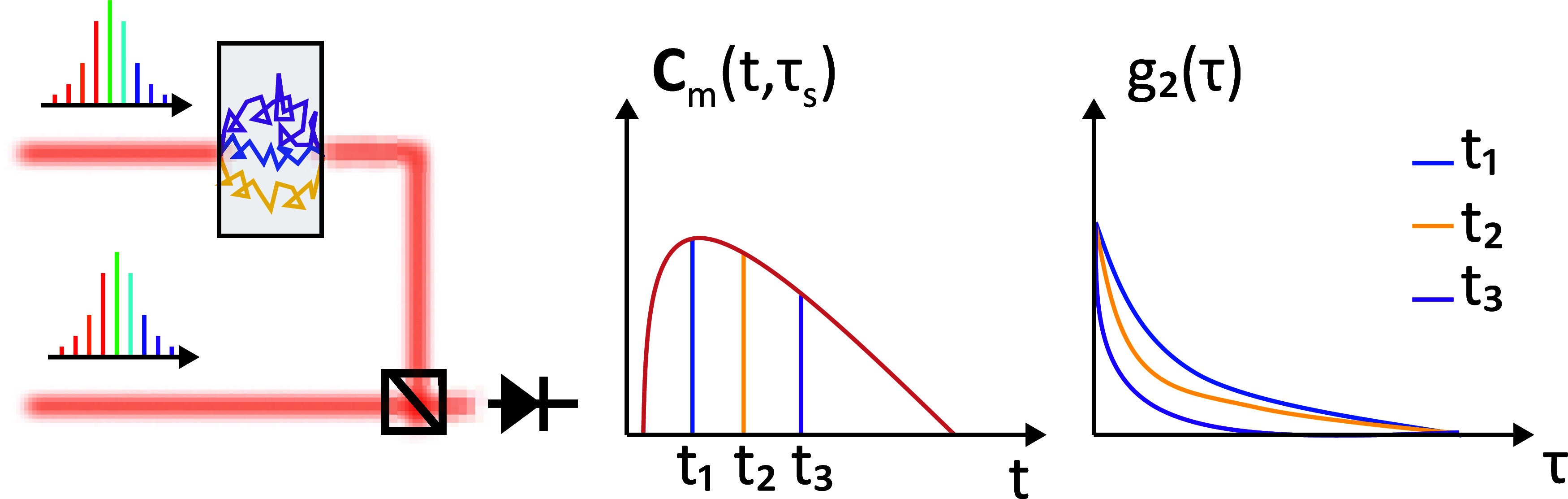

In measuring cerebral blood flow (CBF) noninvasively using optical techniques, diffusing-wave spectroscopy is often combined with near-infrared spectroscopy to obtain a reliable blood flow index. Measuring the blood flow index at a determined depth remains the ultimate goal. In this study, we present a simple approach using dual-comb lasers where we simultaneously measure the absorption coefficient (μ_a_), the reduced scattering coefficient (μ_s_^′^), and dynamic properties. This system can also effectively differentiate dynamics from various depths, which is crucial for analyzing multilayer dynamics. For CBF measurements, this capability is particularly valuable as it helps mitigate the influence of the scalp and skull, thereby enhancing the specificity of deep tissue.

## 1 Introduction

Cerebral blood flow (CBF) serves as a pivotal biomarker in the diagnosis of ischemic stroke.^1,2^ Although computed tomography^3,4^ and magnetic resonance imaging^5,6^ are the gold standards for imaging-based diagnosis, they are hindered by disadvantages such as radiation exposure, high financial costs, and the necessity for patient transportation to medical facilities, thereby reducing valuable time for intervention. Optical techniques to study human physiology have centered around acquiring insights into the propagation of light in diffuse media.^7^ Promising techniques have emerged like near-infrared spectroscopy (NIRS), which leverages the low absorption of tissue in the NIR window to yield reduced scattering coefficients μ_s_^′^ and absorption coefficients μ_a_ by pulsed source (time domain NIRS, TD-NIRS)^8^ or modulated source (frequency domain NIRS, FD-NIRS),^9^ optical coherence tomography (OCT), which is an interferometric imaging technique,^10^ and diffusing-wave spectroscopy [DWS, also called diffuse correlation spectroscopy (DCS) in literature] which use coherent light and study photon correlation to extract blood flow dynamics.^11−13^ Due to the different absorption characteristics of oxyhemoglobin and deoxyhemoglobin, NIRS can infer blood oxygenation levels based on the measured absorption coefficient μ_a_ and additionally assess changes in blood flow. In contrast, OCT determines absolute blood flow using the Doppler effect^14^ but is limited by a low penetration depth of 1 to 3 mm. This limitation arises because conventional OCT detects only single scattered photons, which restricts its ability to measure CBF noninvasively, although recent advancements in OCT that utilize multiple scattering have been shown to enhance image contrast.^15^ On the other hand, DWS employs a separate source-detector configuration to capture multiple scattered photons, offering the advantage of a higher penetration depth of 1 to 3 cm. However, the accuracy of DWS is contingent on the tissue’s optical properties like reduced scattering coefficient μ_s_^′^ and the absorption coefficient μ_a_, which can vary across different tissues and patients.^16^ These are then ascertained with multiple methodologies, including NIRS. Furthermore, these techniques have distinct equipment requirements: DWS necessitates a light source with a long coherence length, NIRS requires a pulsed source or an intensity-modulated source, and OCT requires a stable, broad-bandwidth source or a swept source. Consequently, many experimental setups with complex instrumentation have been developed to integrate aspects of these techniques as they provide complementary information.^17−20^ Extracting all necessary parameters from a single modality is thus extremely essential and an ongoing effort in the light-tissue interaction community. For instance, long coherence length pulsed lasers to combine DWS and TD-NIRS^16,21,22^ have been demonstrated. New interferometric techniques have also demonstrated experiments to extract parameters from a single modality.^23−26^ On the other hand, in the CBF measurement, the flow dynamics in the scalp, skull, and cortex are compounded together, affecting the accuracy of CBF assessment. Increasing the source-detector separation in DWS can enhance specificity to deep flow but results in low photon throughput.^27−29^ An alternative approach involves time-of-flight (ToF)-resolved techniques to analyze photon fluctuations from deeper layers, thereby excluding influences from shallower layers.^16,25,26^

The optical frequency comb (OFC)^30−32^ emerges as a promising solution to these limitations. It functions as a pulsed laser in the time domain, making it suitable for TD-NIRS. In the frequency domain, the OFC consists of numerous discrete, equally spaced narrow lines, which provide a sufficiently long coherence length for DWS. Recently Barreiro et al.^33^ used an electro-optical (EO) dual-comb system to measure the frequency response of a turbid medium and reconstruct the distribution of ToF (DTOF) via inverse Fourier transform. However, the dynamic properties have not been fully explored. Besides, EO combs currently require various high-speed modulators and RF sources and have coherence properties limited by the RF oscillator noise.^34^ In contrast, passively mode-locked lasers can achieve a lower noise and do not require any active modulation schemes, making them an appealing alternative. In the context of dual-comb generation, these advantages can be emphasized by ensuring that the two combs share the same laser cavity.^35^ As well as reducing complexity, such approaches reduce noise as well, since most perturbations to the cavity influence both combs in the same way, leading to a high degree of correlation in the fluctuations of the comb line frequencies. Single-cavity dual-comb sources and applications have become a hot topic as reviewed recently.^36^ Here, we use a spatial multiplexing approach where an intracavity Fresnel biprism is used to generate both combs in the same diode-pumped solid-state laser cavity arrangement.^37^ This approach enables low-noise dual-comb generation while benefiting from the high power and low fundamental noise properties of diode-pumped solid-state lasers. In recent work, we have shown that these dual combs are compatible with coherent averaging and can support repetition rates up to a gigahertz or more.^38^

Leveraging these characteristics, we demonstrate a dual-comb DWS system that simultaneously measures static properties (μ_s_^′^ and μ_a_) and depth-dependent dynamics of a homogeneous turbid medium. By combining an auxiliary arm, we correct the relative timing jitter and relative carrier-envelope phase (CEP) shift for the measurement arm in postprocessing. This approach simplifies traditional dual-comb implementation by avoiding the complexity associated with measuring, controlling, or tracking the absolute optical frequencies of the combs.^39^ Furthermore, we use an etalon to reduce the optical bandwidth in order to boost the signal-to-noise ratio (SNR).^40^ This enables the detection of subtle features and weak signals, which were previously challenging to observe, thereby significantly enhancing the performance of the measurement system, resulting in a temporal resolution of 39 ps. For the first time, as far as we know, we have integrated TD-NIRS and DWS into a single modality using a dual-comb technique. Contrary to the approach taken by,^33^ we measure DTOF directly in the time domain. Our study confirms that the noise of our laser is sufficiently low that we can compensate for its timing and phase fluctuations in postprocessing. This represents an advantage compared to the system reported in^33^ and is very beneficial for dynamic measurements. Additionally, we explore the trade-offs among resolution, measurement speed, and SNR, highlighting how low resolution can lead to distortions in the true DTOF. We also propose strategies to mitigate this limitation.

## 2 Methods

### 2.1 DTOF Measurement

ToF measurements based on dual-comb lasers, such as light detection and ranging^41^ or OCT,^42,43^ use two frequency combs with a delay increment of  each pulse period, thus achieving equivalent time sampling, also referred to as asynchronous optical sampling, where Δ*f*_rep_ is the repetition difference of two comb and  and  are the repetition rate of two combs, respectively. The complex representation of the electric field of the frequency comb is given by
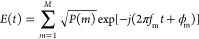
1where *P*(*m*) is the power of the *m*th mode of the comb, *f*_m_ = *f*_ceo_ + *mf*_rep_ is the optical frequency of the *m*th comb mode, *f*_ceo_ is the carrier-envelope offset frequency, , is the repetition frequency, and ϕ_m_ is the phase offset of comb mode *m*.

**Figure 1:**
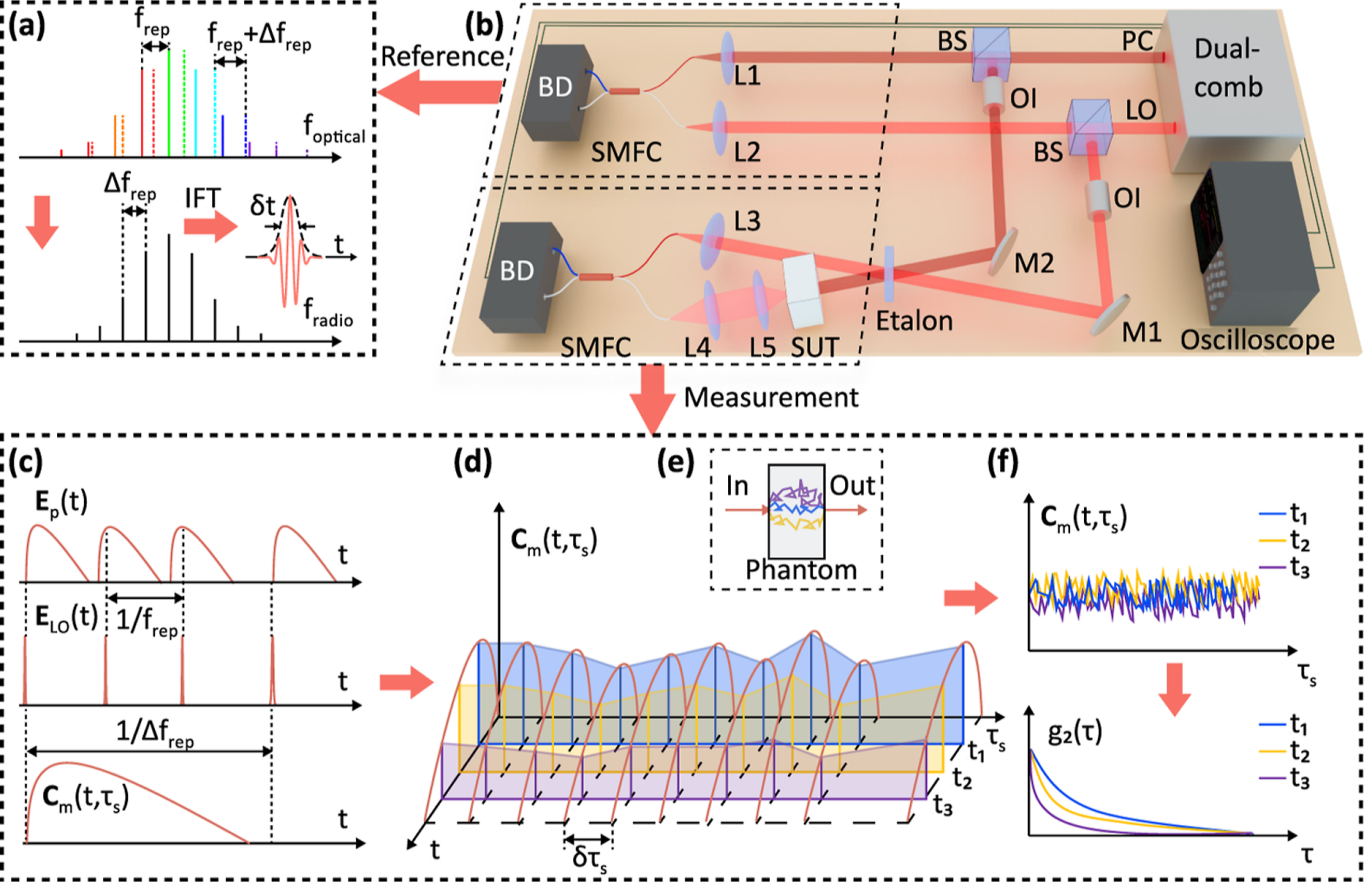
Schematic diagram of dual-comb DCS. (a) Dual-comb spectrum in the optical frequency domain and their interference spectrum in the radio frequency domain. (b) Two dual-comb Mach–Zehnder interferometers work as reference and measurement, respectively; PC-probe comb, LO-local oscillator, BS-beam splitter, OI-optical isolator, L1–L5-lenses, M1–M2-mirror, SMFC-single mode fiber coupler, SUT-sample under test, and BD-balanced detectors. (c) The ToFs of scattered photons obey a certain distribution (DTOF) which is a function of μ_s_^′^ and μ_a_, and the LO scans them with step  and finishes one cycle scanning in 1/Δ*f*_rep_, *E*_p_(*t*) and *E*_LO_(*t*) are the electric fields of probe comb and LO, respectively. (d) The LO periodically scans the reemitted photons from each pulse at an interval δ_τ_s__ = 1/Δ*f*. The interference signal fluctuates due to the dynamics of the scatterers. This rate of fluctuation varies for different ToFs. (e) Different scattering paths in the sample. (f) Trace the intensity of different ToFs and do autocorrelation, a longer ToF owns a higher decay rate. Note: all carrier oscillations are omitted in (c,d).

In the laser system used here (Figure 1b), both combs are emitted from the same spatially multiplexed Yb:YAG dual-comb laser cavity and have central wavelengths of 1030 nm. The laser has a 247 MHz repetition rate and is similar in design to the one discussed in.^44^ After the beam splitters, they are split into two arms. One arm serves as a reference where the probe comb and the local oscillator (LO) combine directly via a single-mode fiber coupler (SMFC) and are then detected by a balanced detector (BD) to monitor the status of the laser. The cross terms of comb modes belonging to the same comb and that belonging to the other comb whose frequency difference is higher than *f*_rep_/2 disappear after applying bandpass filters. As a consequence, only the cross terms of adjacent comb modes belonging to two different combs are considered. The optical frequency is downconverted to *f*_0_ + *m*Δ*f*_rep_, thus generating a new comb in the radio frequency domain that the BDs can detect (Figure 1a), where *f*_0_ is an effective start frequency of the RF comb. BDs remove the DC component of the interferogram yielding
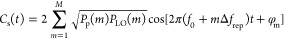
2where *P*_p_(*m*) and *P*_LO_(*m*) are the power of the *m*th mode of corresponding source and φ_*m*_ is the phase offset for the *m*th comb mode.

**Figure 2:**
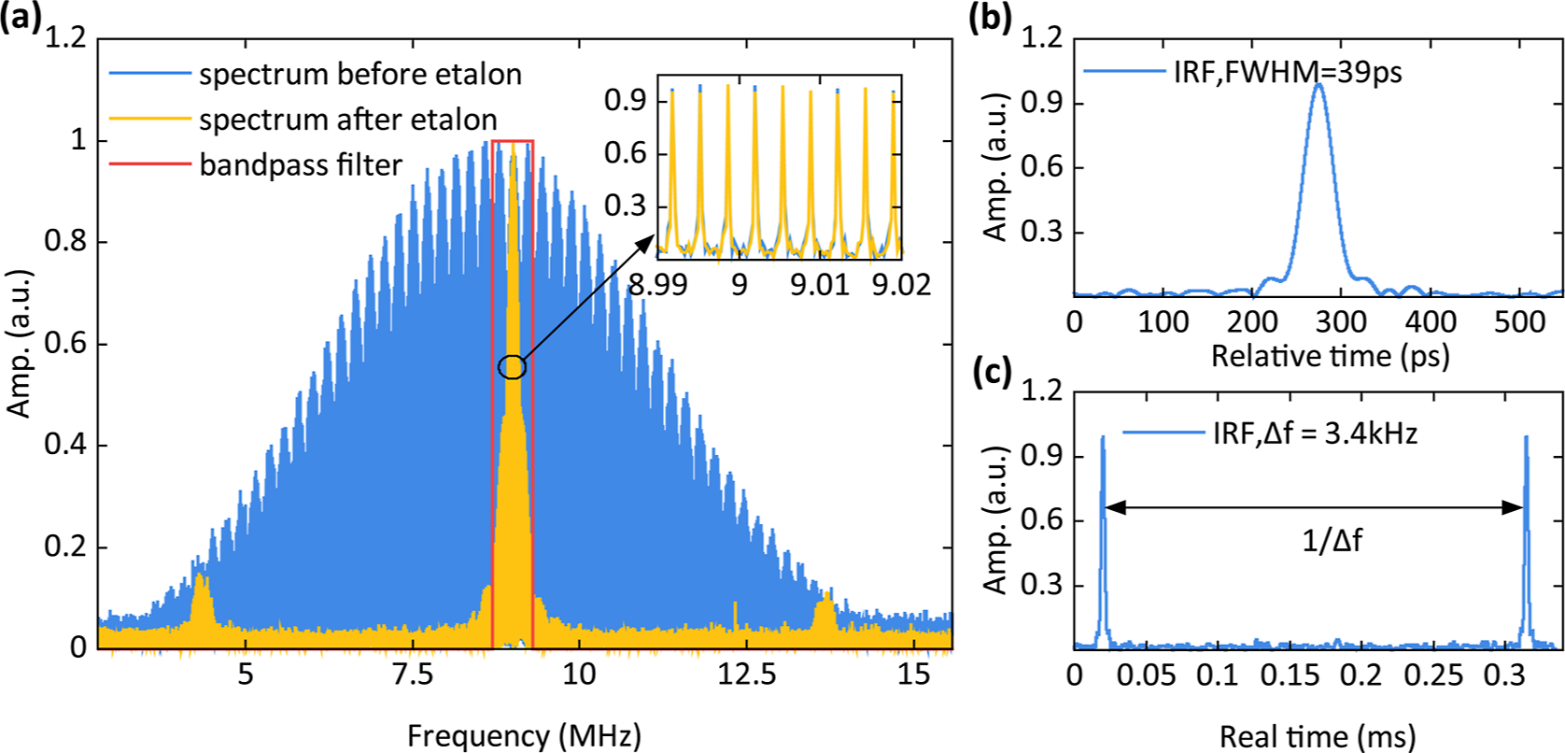
Laser spectrum, instrument response function (IRF), and the refresh rate. (a) Spectrum of the radio frequency comb, the blue line represents the spectrum before etalon, the yellow line represents the spectrum after etalon, and the red line represents the bandpass filter used for postprocessing; the inset shows the zoom-in of the central spectrum. (b) The envelope of interference signal in the reference arm is the IRF here. (c) Adjacent pulses in the time domain, the refresh rate indicates the measurement speed. Note: *t*_r_ = *t*/(*f*_rep_/Δ*f*_rep_), where *t* is the real time and *t*_r_ is the relative time between two combs.

In the measurement arm, an etalon is placed before the sample to decrease the illumination power. By doing so, we keep the average power of individual comb modes as high as possible and reduce the detection bandwidth, thus raising SNR,^40^ and the detailed description is shown in Section 2.3. Two side peaks owing to the short etalon free spectral (around 1.5 nm) are filtered out by a digital bandpass filter in postprocessing (Figure 2a). After the etalon and bandpass filter, the optical power of the probe comb is around 100 mW. In ref,^45^ a maximum permissible exposure (MPE) of 103 mW was reported with a 1 mm diameter beam and 1064 nm light. Our configuration closely corresponds to this MPE (100 mW average power and 1030 nm wavelength). On the other hand, the power of the LO is set to 3 mW, which is significantly higher than the power of the diffused photons collected. By leveraging the heterodyne gain, such weak signals can be detected and achieve shot-noise-limited SNR. In the end, 23.27 GHz FWHM optical bandwidth is utilized to give 39 ps characteristic temporal resolution (Figure 2b), and the repetition difference (1/Δ*f*_rep_) is set to 3.4 kHz, thus resulting in 1/Δ*f*_rep_ = 294 μs decay resolution (Figure 2c).

Photons emitted from the probe comb undergo multiple scattering events within the sample, following various paths, as depicted in Figure 1e. This process leads to a distribution of ToF for the photons, known as the DTOF. The DTOF of photons in a turbid slab is described in,^46^ where the pulse laser is modeled as a delta function, and the intensity of their ToFs is derived. Because of a slight repetition frequency difference between the probe comb and the LO, the LO interferes with different portions of scattered photons each period with step , and after 1/Δ*f*_rep_, LO and PC have a maximum delay difference of  and thus overlapping at the start of the next cycle, as shown in Figure 1c. The cross term of two combs in the measurement arm is given by
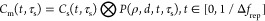
3where τ_s_ is a slow time scale representing the interferogram period, and it therefore has discrete values of *n*/Δ*f*_rep_, *t* is the absolute time and can be transformed to relative time *t*_r_ by *t*_r_ = *t*/(*f*_rep_/Δ*f*_rep_), *P*(ρ, *d*, *t*, τ_s_) is the DTOF of photons, ρ is the horizontal distance between source and detector, *d* is the thickness of the sample, and ⊗ denotes the convolution.

Direct deconvolution of *C*_m_ and *C*_s_ cannot accurately yield *P*(ρ, *d*, *t*, and τ_s_) due to the presence of high noise. In addition, transforming *C*_m_ to the frequency domain and doing demultiplication are not effective either as the noise overwhelms the comb modes. However, we have discovered that by averaging the envelope of *C*_m_, it is possible to successfully reconstruct *P*(ρ, *d*, and *t*).

**Figure 3:**
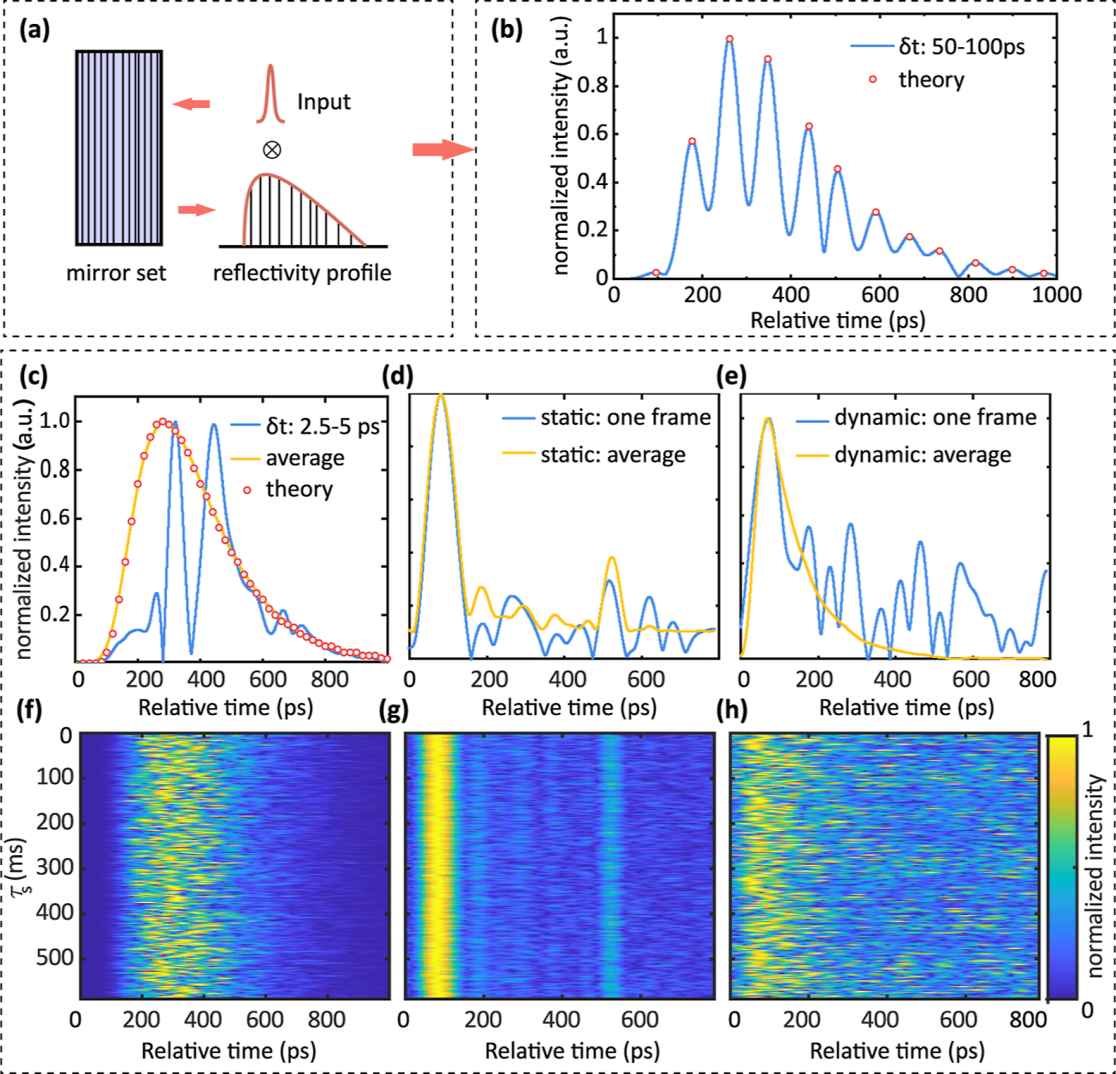
Influence of low resolution: a simulation, static sample measurement, and dynamic sample measurement. (a) Schematic diagram of simulation. The scattering sample is modeled as a mirror set with a reflectivity profile. (b,c) 39 ps-fwhm IRF convolved with reflectivity profile with 50–100 and 2.5–5 ps ToF separation respectively, and the red dot represents mirror reflectivity. (d,e) One interferogram envelope of a static and a dynamic sample and 2000 interferogram averaging results. (f–h) Interferogram envelope over 2000 frames of the simulation, static sample, and dynamic sample. The horizontal axis represents ToF, the vertical axis represents the time lag of different measurement periods, and the color bar represents normalized intensity.

To show how envelope averaging constructs the DTOF, a simulation is conducted. We consider scattering photons with different ToFs as being reflected from a distribution of virtual mirrors located at different depths in the sample (Figure 3a). The reflected signal is obtained by the convolution of *C*_s_(*t*) and *P*(ρ, *d*, *t*, and τ_s_), where we set ρ = 0, *d* = 10 mm. To mimic dynamics, the ToF distribution is reinitialized with a different set of virtual mirror positions following the same statistical distribution. The fwhm of *C*_s_(*t*) is 39 ps (relative time).

Initially, when δ*t* (the separation of reflected photons) ranges between 50 and 100 ps, as shown in Figure 3b, the resolution of the dual-comb system is sufficiently high to differentiate between the reflections from the different mirrors. The peaks of the envelope of *C*_*m*_(*t*) align well with the theoretical predictions. Subsequently, when δ*t* is reduced to 2.5–5 ps (Figure 3c), the mirrors can no longer be resolved by the dual-comb system. This setup differs from a typical low-coherence pulsed laser system as photons reflected from adjacent mirrors are coherent and thus interfere with each other, leading to artifacts (as illustrated by the blue line in Figure 3c). However, by averaging 2000 randomly generated interferogram envelopes (Figure 3f), these interference effects can be mitigated. The result (as shown by the yellow line in Figure 3c) aligns closely with the predefined set *P*(ρ, *d*, *t*, and τ_s_).

We also show the result of a static sample with 9.5 cm^–1^ μ_s_′ and 0.05 cm^–1^ μ_a_, which is made of glass spheres and polydimethylsiloxane and cured at 75 °C.^47^ Since the scatterers remain stationary, the ToF separation remains unchanged, and the interferogram of each frame remains stable except for some white noise (Figure 3g). Consequently, the averaged results show no significant difference compared to a single frame (Figure 3d). Conversely, for a dynamic sample (Intralipid phantom), the moving scatters cause ToF separation to keep changing; thus, the interferogram of each frame is different. By averaging, the true DTOF can be constructed (Figure 3e), which is compared with diffusion theory in Section 2.1. The small peak around 500 ps in Figure 3d,g comes from spurious reflection in the setup or inside the laser. In the dynamic phantom measurement, we included an additional fiber to introduce a delay such that this peak is temporally separated from the signal of interest near a relative time = 0.

### 2.2 Dynamics Measurement

To start the measurement of the dynamic sample, the CEP shift in the measurement arm must be clarified. The dynamics of particles inside the sample cause a phase shift of each scattering path; as a result, the interferogram of the two combs fluctuates. In biological tissue, red blood cells (RBCs) from different layers have different velocities, and in the homogeneous turbid medium, the scattering event number varies at different ToFs; thus, the phase shift velocity varies. This can be distinguished by tracing the fluctuation at different ToFs and applying autocorrelation to them, as shown in Figure 1d,f. However, because the laser is in the free-running mode, it exhibits small but timing jitter and optical phase fluctuations of the dual-comb interferograms, which are mainly from mechanical/acoustic noise and pump diode fluctuations. By accounting for these fluctuations via a reference channel, much more information can be obtained from the dynamic measurement. In the dual-comb spectroscopy phase, timing errors can be corrected digitally to obtain coherently averaged signals.^48^ Usually, those errors are easier to correct for a high repetition difference as the higher repetition difference corresponds to a higher tracking speed. In this work, by using a mechanically robust prototype implementation of spatial multiplexing, we are able to satisfy the condition for coherent averaging at <1 kHz Δ*f*_rep_.^38^ To compensate for the relative timing jitter and relative phase shift in the measurement arm, we built a reference arm to monitor those parameters and then correct them for the measurement arm. After this, the path-dependent intensity autocorrelation is calculated by
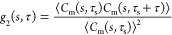
4where *s* = *n*_m_*ct* is the path length of scattering photons, *n*_m_ is the refractive index of the medium, and *c* is the light speed under vacuum. Furthermore, the intensity autocorrelation is related to field autocorrelation by the Siegert relation: *g*_2_(τ) = 1 + β|*g*_1_(τ)|^2^, where β ranges from 0 to 1 and accounts for the measured speckle number. According to DWS,^49^ the path-dependent normalized field autocorrelation function is denoted by
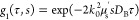
5where *D*_B_ is the Brownian motion coefficient, μ_s_′ is the reduced scattering coefficient of turbid medium, and *k*_0_ is the wavenumber of the light in the medium. In biological tissue, photons are scattered by RBCs in different vessels. Although RBCs exhibit laminar flow within a single vessel, their dynamics resemble Brownian motion when measured using DWS.^50^ Combined with the blood volume fraction (denoted as α), the blood flow index α*D*_B_ can be calculated.

### 2.3 SNR

In a dual-comb interferometer, the peak value of the interference signal is given by
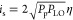
6where *i*_s_ (A) is the current after the BD, η (A/W) is the responsivity of the BD, and *P*_p_ (W) and *P*_LO_ (W) are the average power of transmitted probe comb and LO, respectively. The laser intensity noise is negligible due to the ultralow noise of the laser utilized here and the balanced detection canceling out common noise; therefore, only shot noise and detector noise are considered here
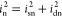
7where shot noise  (*P*_p_ ≪ *P*_LO_), *B* is the detection bandwidth, and *q* is the electron charge. The detector noise ,  is the noise-equivalent power of the detector. When increasing the power of the LO, *i*_sn_^2^ ≫ *i*_dn_^2^; thus, the shot-noise limited SNR can be achieved
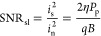
8

For a given power of probe comb, SNR_sl_ is limited by detection bandwidth, which is related to temporal resolution by δ(ToF) ∼ λ^2^/Δλ, and λ is the central wavelength and Δλ is the optical bandwidth and is mapped into the radio frequency domain in dual-comb measurement. The detection bandwidth is given by *B* = *N*Δ*f*_rep_, and *P*_p_ can further be written by *P*_p_ = *NP*_*m*_, where *P*_*m*_ is the power of a single comb mode and *N* is the number of modes after the bandpass filter (rectangular spectrum is assumed here), then
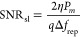
9

For a given measurement speed (Δ*f*_rep_), *P*_*m*_ determines the shot-noise limited SNR and is limited by MPE.

In general, there is a trade-off in dual-comb DWS between temporal resolution, measurement speed, and SNR. This trade-off should be considered carefully when analyzing dynamics in deep tissue since stronger scattering leads to a reduced wavefront overlap of the scattered photons with the LO comb photons. Here, the repetition difference of the dual-comb laser is set to 3.4 kHz (Δ*f*_rep_ can be adjusted to an arbitrary value, and the maximum Δ*f*_rep_ depends on the internal optical configuration inside the cavity and can be tens of kHz), and an etalon is employed to narrow down the optical bandwidth; meanwhile, a digital bandpass filter is applied to the RF comb spectrum. By doing so, there are several benefits: (1) the SNR is proportional to the measurement time, so by reducing Δ*f*_rep_ we obtain a large SNR from a single period, without the need to coherently average multiple periods. (2) The etalon filters other comb modes and reduces the power of the PC and the LO. The power reduction of PC makes sure that it is lower than MPE and meanwhile keeps the power of individual comb modes as high as possible, which gives us high SNR (as shown in eq 9). (3) The bandpass filter fits the RF comb spectrum and enables us to remove noise in the absence of the signal, which increases shot-noise limited SNR (as shown in eq 8).

## 3 Results

### 3.1 Static Properties

To validate the ability of the DTOF measurement of the dual-comb system, we build a set of 10 mm thickness homogeneous samples, which consist of 6 mL of pure water and 0.2–2 mL of 20% Intralipid emulsion (Sigma-Aldrich, 0.2 mL per step), and placed them in the measurement arm.

**Figure 4:**
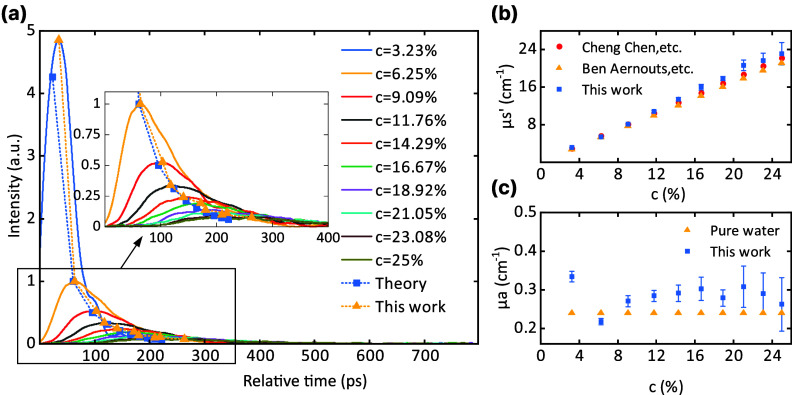
Measured DTOF, reduced scattering coefficient (μ_s_^′^) and absorption coefficient (μ_a_) of samples with different Intralipid concentrations. (a) Measured DTOF curves of the sample with different concentrations. The peak points and their arrival times are compared with that of diffusion theory. (b) DTOF curves are fitted to extract the reduced scattering coefficient, compared with the value in the literature.^51,52^ (c) Comparison of the extracted absorption coefficient with pure water.^53^

During the measurement, we removed the sample first to obtain the peak position of the interferogram. Then, we calculated back to determine the starting point of DTOF using the thickness of the sample. The DTOF is acquired by averaging the envelope of the interferogram and deconvolution with IRF. Figure 4a shows the DTOF of 10 samples (10 measurements per sample), and their intensities are normalized by the *c* = 6.25% sample. As the concentration increases, the intensity decreases due to scattering, and the arrival time of peaks is delayed. To compare with the diffusion theory, the theoretical μ_s_′ of 20% Intralipid^52^ and μ_a_ of pure water^53^ at 1030 nm (μ_a_ of Intralipid is smaller than water) are referred. For samples with different concentrations, the reduced scattering coefficient is calculated by μ(*c*)_s_′ = *c*μ_s_′, and μ_a_ remains the same. By substituting those values into *P*(ρ, *d*, *t*, τ_s_),^46^ the peak value and arrival time of different samples are predicted and compared with this work (Figure 4a). Except for the case where *c* = 3.23%, which does not align with diffusion theory due to low scattering, the other cases largely conform to diffusion theory. Furthermore, we fit the DTOF curve and extract μ_s_′ (Figure 4b) and μ_a_ (Figure 4c) by *P*(ρ, *d*, *t*). The measured μ_s_′ show high alignment with the results in^51^ and.^52^ μ_a_ shows a discrepancy in the first sample but close to the result in^53^ when concentration rises.

### 3.2 Dynamic Properties

Although the single cavity dual comb owns the merits of ultralow noise, environmental disturbances such as vibrations lead to jitter in the timing and CEP of the interferograms. In the measurement arm, the phase shifts that come from the dynamics of the sample are compounded by the laser itself. Therefore, it is necessary to compensate for those relative phase shifts that come from the laser first.

**Figure 5:**
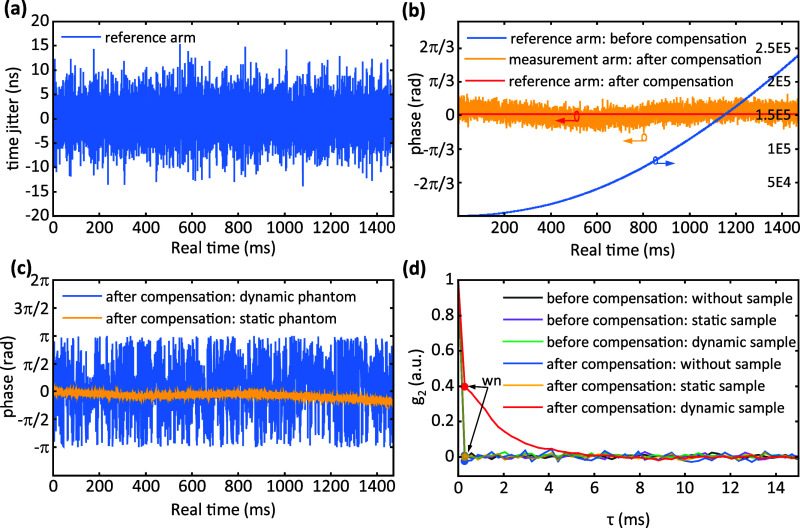
Measurement of the timing and phase noise properties of the interferograms and their correction. (a) Relative timing jitter between the two combs (equivalently, timing jitter of the interferograms scaled down to the optical domain by the factor Δ*f*_rep_/*f*_rep_). (b) The unwrapped relative CEP shifts before and after compensation in the reference arm, and that of the measurement arm after compensation without a sample. (c) CEP shifts of the interferogram after compensation in the measurement arm with the static and dynamic sample. (d) Comparison of the second-order autocorrelation before and after compensation with air, static sample, and dynamic sample, and wn represents white noise drop.

Owing to the high SNR in the reference arm, the relative timing jitter and the relative CEP shift can easily be extracted and compensated by using the method^38^ described earlier. Figure 5a shows the relative timing jitter of the pulses before compensation in the reference arm, which is the main reason for the relative CEP shift (Figure 5b). The unwrapped relative phase shift before compensation grows quadratically (blue line in Figure 5b) while it is flat (equals 0, red line in Figure 5b) after compensation in the reference arm. After they are compensated to the measurement arm where the sample is removed, the relative CEP is much lower than 2π (yellow line in Figure 5b). Moreover, we also show the compensation result of a static sample (yellow line in Figure 5c) and dynamic sample (blue line in Figure 5c), where the former is close to 0 but the latter bounds between −π and π, which reveals that only movement of scatters induces phase shift after compensation. After tracing the peak point of the interferogram in air, and certain ToF in the static and dynamic sample, the second-order autocorrelation (*g*_2_) is calculated (Figure 5d). For air and static samples, *g*_2_ is expected to be flat; however, in our observations, it drops to zero immediately before and after compensation. This phenomenon occurs because the standard autocorrelation function employed here first subtracts the mean value of the signal, leaving only white noise (comprising shot noise and electronic noise). Since white noise does not exhibit any correlation, we see a drop. For dynamic samples, the drop observed before compensation is similar and can be attributed to laser noise. After compensation, we first notice a drop due to white noise, followed by a decay curve. This curve provides dynamic information about the sample, i.e., Brownian motion coefficient of the sample.

**Figure 6:**
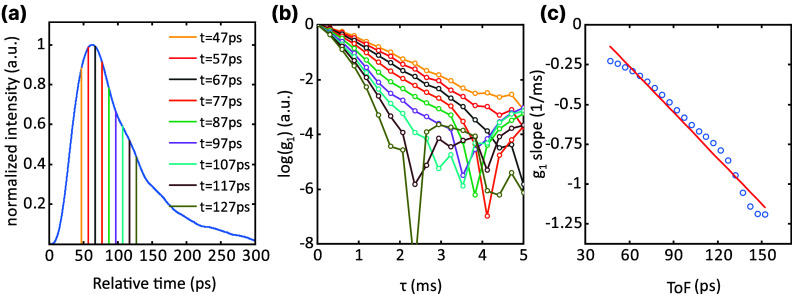
Traced intensity fluctuation of different ToFs, and the first-order autocorrelation used to extract the Browian coefficient. (a) DTOF curve of Intralipid sample. (b) *g*_1_ Of the different decay rates at different ToFs. (c) The decay rate increases linearly with ToFs.

To reduce the Brownian motion speed that can be measured by this system’s refresh rate, we mixed 18 mL of 20% Intralipid emulsion with 10 mL of glycerin, whose viscosity is higher than pure water. After the relative timing jitter and relative phase shift are compensated, the movement of scatters is the only cause of the intensity fluctuation. μ_s_′ is measured by using the method described in Section 3.1 to be 5.6 cm^–1^ (Figure 6a) for a homogeneous Intralipid sample where only Brownian motion exists. Figure 6a shows how ToF is distributed in the Intralipid sample. The vertical lines indicate specific relative times, and in Figure 6b, we show the autocorrelation function *g*_1_(τ) for these relative times. As expected, components with a larger ToF (i.e., a larger relative time in Figure 6a) exhibit a faster decay of *g*_1_(τ) with respect to real-time τ. From eq 5, the decay slope is proportional to the ToF *t* according to d[ln(*g*_1_(τ))]/dτ = −2*k*_0_^2^μ_s_′*sD*_B_ = −2*k*_0_^2^μ_*s*_′*D*_B_*vt*. To quantify this dependence, in Figure 6c, we show the decay rate as a function of ToF. The figure confirms the linear relation, and we can infer a Brownian motion coefficient *D*_B_ = 0.27 × 10^–12^ m^2^/s.

## 4 Conclusions and Discussion

The dual-comb technology, known for its ultrastable frequency and broadened bandwidth, has been widely utilized in absorption spectroscopy and precise metrology. However, its practical application has been limited due to the high system complexity. We have demonstrated a dual-comb system with a simpler configuration specifically for medical applications, offering three significant advantages.

First, the laser’s novel structure enables high power output and low relative intensity noise without the need for a laser amplifier, thereby avoiding the amplified spontaneous emission noise typically associated with such amplifiers. The single-cavity design produces two independent pulse trains with significant noise correlations, eliminating the locking electronics requirement, as mutual coherence is obtained passively. Additionally, the 250 MHz repetition rate laser is rather simple and compact and can avoid DTOF overlap for clinic requirements. Furthermore, the combination of high power and an etalon significantly enhances the SNR. The ultralow noise level allows for the measurement of sample dynamics using a coherent averaging algorithm at a repetition rate difference as low as 3.4 kHz.

Second, our dual-comb DWS system can simultaneously measure the absorption coefficient (μ_a_), the reduced scattering coefficient (μ_s_′), and dynamic properties. While averaging is necessary to obtain the DTOF, μ_a_ and μ_s_′ typically do not fluctuate rapidly over time. In our experience, averaging over approximately 2000 frames (0.59 s) is sufficient to construct DTOF, although this may vary depending on the μ_s_′ and μ_a_ of the sample. Additionally, our findings offer insights for dual-comb OCT, particularly that a sufficiently high laser bandwidth is crucial to avoid artifacts caused by interference between adjacent layers. Further research using an appropriate frequency comb laser with an emission spectrum spanning an isosbestic point of blood will allow the distinct absorption characteristics of oxyhemoglobin and deoxyhemoglobin to be measured. This will enable the quantification of blood oxygenation from μ_a_. Moreover, by combining this with blood flow measurements, we can also determine the blood metabolism in the future.

Lastly, this system effectively differentiates dynamics from various paths (or TOFs), crucial for analyzing multilayer dynamics as photons that traverse deeper layers exhibit longer ToFs. For CBF measurements, this capability is precious as it helps mitigate the influence of the scalp and skull, thereby enhancing the specificity to deep tissue and improving the accuracy of thrombus localization for ischemic stroke. However, the specificity to deeper layers is also dependent on the SNR. Given the low duty cycle of pulsed lasers, optimizing the bandwidth, measurement speed, and illumination power is essential to achieve a performance comparable to that of continuous lasers. Enhancing the SNR will be a primary focus of our future work. We also see that the use of a broadband coherent pulsed source will remove the need for many light sources or spectral scanning needed to access optical properties in biological media.
